# An End-User’s Personal Perspective on the Need of Consumer Involvement in Research

**DOI:** 10.1007/s11606-021-07125-5

**Published:** 2022-03-29

**Authors:** S. M. Ferry, M. Kristi Henzel, Kath M. Bogie

**Affiliations:** 1 Erie, PA USA; 2 Louis Stokes Cleveland VA Medical Center, 10701 East Blvd, Cleveland, OH 44106 USA; 3 Case Western Reserve University, 10900 Euclid Ave, Cleveland, OH 44106 USA

## Abstract

There is growing understanding that the consumer’s voice in research needs to be stronger. Translational research studies need consumer inclusion in order to be effectively implemented. This narrative article provides the perspective of a Veteran with spinal cord injury (SCI) who is an active member of several study teams and serves as a Consumer Advocate, providing the voice of the person with SCI. Factors that drive people to develop new research ideas are considered. Consumer involvement offers helpful insight into project outcomes that are valuable to the end-user. It is also recognized that data can be interpreted in several different ways depending on the observer. Including the consumer in a research project enables another interpretation, creating a more complete evaluation. Participating in health research is becoming a new standard for persons with many different illnesses and diseases. Greater things are accomplished by physicians, healthcare scientists, engineers, and healthcare consumers interacting together to increase both the quality of research projects and the quality of life for everyone involved, especially the person with the disorder. There will be more acceptance of ideas or projects when consumers are involved from the early steps and learn how the process works from beginning to end.

## Narrative Main Body

The lead author is a Veteran with a spinal cord injury (SCI) and an active member of the Paralyzed Veterans of America (PVA). He experienced a traumatic SCI in October 2014. He has been an active participant and advocate for the voice of the consumer in research studies since his injury and he has served as a Consumer Reviewer for the Congressionally Directed Medical Research Program. He is currently the Consumer Advocate on two technology development projects based at the Louis Stokes Cleveland VA Medical Center. The project led by Dr. Kath Bogie is developing a low-cost point-of-care sensor which enables a rapid blood test for pressure injury risk [1]. The project led by Dr. Kristi Henzel is focused on reducing the incidence of inadvertent lower limb injuries for wheelchair users through development of a smart wireless sensor system that captures foot position in real time [2].

The lead author was introduced to Dr. Bogie and her research project during his initial rehabilitation stay at the Cleveland VA. He was very intrigued as she thoroughly explained that there would be some invasive biopsy requirements as well as pressure mapping of his seating position in her laboratory. At first, it sounded possibly too involved but, after a short thought process and some spinal research on his own, he realized that he may be facing a grim future involving pressure injuries. He wanted to enroll in the research project to avoid that scenario for himself, as well as peers. Several biopsies, CT scans, and laboratory visits were accomplished over the next four years. As the project progressed, Mr. Ferry was asked to be on the team as a consumer advocate.

There is a growing understanding that the consumer’s voice needs to be louder than it ever has been before. In today’s world, the most successful research programs are including the end-user and the consumer. Many of the initial scientific steps of most projects may not be familiar to most advocates but the new concept involving listening and participating throughout the whole process is a motivating factor. Once the projects reach the stage where they can be discussed with peers and other community professionals, the advocate is able to step up their responsibility and speak with significant knowledge of the whole process and not just the result. Personal project knowledge will encourage support.

There seems to be several common factors that drive people to develop new research ideas. Public-spirited ambition and personal drive seem to play a key role. Not every research professional has the same personal drive to think of ways to improve the quality of life for the people they are studying. Some research is initiated by people who have a strong internal drive to improve society and, through their personal contributions, will stand out among their peers. Many of the wonderful people who choose a clinical career path seem to be instilled with an internal ambition that also seeks to support and lead research projects to help ease illnesses. Many healthcare scientists and engineers also exercise an internal ambition to find out why things happen, how to prevent them from happening, or how to make things better. They are among the people who have personal drive to improve the quality of life for others as a core of who they are.

It is important to recognize that ambition also comes from consumers, the people who live day to day with illnesses and diseases, to improve their quality of life. The consumer perspective brings beneficial factors, data, and personal lived experience to the table. As a bit of perspective, most people would not go to a famous chef to buy a used car. This simple concept shows a commonsense reason why research projects need involvement from the consumer. Having the insight from the consumer will also heighten the intrigue and excite the minds of other people with the same condition and encourage them to participate in a research project. Having a consumer as a team member in a research project may not always directly benefit that person but will always aid in the overall success of a project. Potential research participants are undoubtedly more likely to be receptive knowing that a peer contributed to something being introduced to them.

Many healthcare interventions are implemented due to research projects that are driven by data. However, data can be interpreted in several different ways depending on the observer. A doctor could review the data and see clinical research ideas. A healthcare scientist or engineer looks at the same data and envisions a technological or informatics-based solution. The consumer evaluates a completely different situation than the scientist or doctor and often sees a need for different aspects of research to be prioritized. The consumer perspective brings the personal viewpoint. As an advocate, the lead author can identify scenarios causing pressure injuries that may be outside of the commonly accepted risk of just sitting in a wheelchair. Personal experiences of pressure injuries and foot displacement that were not identified were suggested through interpretation of an end-user advocate. The consumer interprets data in the context of personal experience and how the research will directly impact their quality of life and function. Being involved in research project planning allows consumers to communicate at an understandable level to the community in support of the project. In Dr. Bogie’s project, one interpretation of success may be the ability to read the biomarkers to positively identify the desired outcome. Having an advocate involved in the project helps to accomplish a successful interpretation targeting a process that is accessible and manageable to all the people it would benefit. In Dr. Henzel’s project, it may take an advocate to identify more scenarios of foot displacement than occur to scientists or non-wheelchair users.

Some research projects are initiated with a goal of personal gain. Even if you are a medical professional initiating research due to a personal illness, you are going to want more consumer inclusion. Sometimes certain professional fields house a different environment for a person with the disease than a person with the same disease in a different region or job field. There are many benefits to working in a group that shares ambition. As an advocate for the two projects mentioned, the lead author will be addressing peer groups to promote the success and personal gain from their outcome. Being able to speak as someone involved from the incredibly early stages will build better trust and acceptance.

Participating in health research is becoming a new standard for persons with health problems. The lead author of this article can confirm consumer participation in a research project builds self-esteem, positive attitude, and the internal feeling of worth. When a person is told that they need to try something because a group of people sat in a laboratory and decided it was best for them, it may not feel so appealing. However, when they know that a peer just like them helped develop the project, there is a better willingness to try things and help the research succeed. Personal interaction with physicians and scientists on a project about a topic that relates to oneself as an end-user has a huge impact on a person’s willingness to engage and advocate for that project. The two projects that involve the lead author directly contribute to a better quality of life.

Social media also plays a factor in feeding the minds of the consumers who need the improvement in their quality of life. Peer groups communicate at a much faster rate than traditional research publications and presentations. They communicate in more accessible forums than peer-reviewed journals and professional conferences. Positive perspectives will be broadcast wide and fast by consumers who are involved in projects that impact their quality of life.

The brilliant idea of physicians, healthcare scientists, and consumers interacting together to target a specific goal will increase the quality of research projects and the quality of life for everyone involved. These collaborative projects should no longer just be about improving healthcare for the consumer but working toward that goal with the person with the disease. There will be more acceptance to an idea or project when consumers are involved from the early steps and learn how the process works from beginning to end. In the past, the knowledge of the behind-the-scenes work that goes into starting and running a research project has not been well identified for the end-users. Transparency is an essential key word for our future and will bring only positive things to new research projects as they develop. When a consumer or consumer group is involved from the early stages of a project, there will be greater positive impact evolving through the input of another link in the chain that is essential to building a stronger, more knowledgeable, and inclusive society.
